# The epizootic situation of cattle moraxellosis in several economic entities of the Republic of Kazakhstan

**DOI:** 10.14202/vetworld.2021.1380-1388

**Published:** 2021-05-30

**Authors:** N. P. Ivanov, F. A. Bakiyeva, A. M. Namet, R. S. Sattarova, B. Zh. Issakulova, N. Zh. Akmyrzayev

**Affiliations:** Department of Epizootological Monitoring and Risk Assessment of Bacterial Diseases of Animals, “Kazakh Scientific Research Veterinary Institute” LLP, Almaty, Republic of Kazakhstan

**Keywords:** distribution, epizootology, import of livestock, moraxellosis, pathogen

## Abstract

**Background and Aim:**

Infectious bovine keratoconjunctivitis (IBK; conjunctivitis) is a widespread eye disease in cattle. In 1960, the Gram-negative bacillus *Moraxella bovis* was discovered as one of IBK’s etiological causal agents. This study aimed to clarify the epidemiological (epizootic) situation of cattle moraxellosis in the Republic of Kazakhstan. The study also maps the quantitative and qualitative indicators of the epidemiological (epizootic) process to develop more effective antiepizootic measures.

**Materials and Methods:**

We clinically examined both imported and local cattle species based on existing epidemiological (epizootological) units of economic entities in Kazakhstan. Then, we selected biomaterials for laboratory tests to screen for moraxellosis.

**Results:**

We clarified the epizootic situation for moraxellosis of cattle in Kazakhstan using data from the Meat Union of Kazakhstan, veterinary reports from the Veterinary Control and Surveillance Committee of the Ministry of Agriculture, Kazakhstan, and our results, obtained during visits to farms in the various regions of the republic.

**Conclusion:**

Based on the data of the conducted studies, we developed epizootic visualization maps with quantitative indicators of the cattle moraxellosis epizootic-transfer processes within various regions of Kazakhstan for 2019. The data obtained from studies of the properties of the isolated cultures compared with the characteristics of reference strains gave grounds to classify them as belonging to the *Moraxella* genus.

## Introduction

Infectious bovine keratoconjunctivitis (IBK; conjunctivitis) is a widespread eye disease in cattle, affecting both dairy and beef breeds worldwide. IBK’s clinical signs include corneal ulceration, corneal edema, eye pain, photophobia, and lacrimation. In the worst case, the cornea can rupture, leading to permanent blindness. In 1960, the Gram-negative bacillus *Moraxella bovis* was discovered to be one of IBK’s etiological agents [1]. IBK is associated with eye ulcers and weight loss of up to 13.6 kg [2,3]. Since IBK can affect up to 30% of calves in a herd [2], it is a common, economically important disease, leading to decreased productivity of cows and calves [4].

*M. bovis*, a Gram-negative, aerobic, oxidase-positive diplococcus that causes eye disease in cattle [5,6], is colloquially known as conjunctivitis, new forest eye, or pinkeye [7]. *M. bovis* was first isolated from cattle with conjunctivitis in 1915 in Bengal, India [8]. The earliest work describing bacterial organisms with IBK is by Billings [9]. IBK can be transmitted in various ways through direct contact from cattle to cattle, while flies act as carriers [10] during grazing, etc. [11]. Similarly, increased infestation occurs in summer and autumn, correlated with increased sunlight and fly populations. Therapeutic and prophylactic measures against IBK include the use of antimicrobial drugs and vaccines. However, the low efficacy of the vaccine hampers prevention. Genetic and antigenic variations between *M. bovis* strains and the putative presence of other microorganisms explain the pathogenesis of this disease [12].

One of the widespread diseases manifested by damage to the organs of vision in cattle is IBK. Bacteria of the *Moraxella* genus cause this disease, which is registered in many countries globally, including in the Republic of Kazakhstan (RK) [13]. In RK territory, in the imported beef cattle, there were eye lesions. In 2016, this disease was identified as infectious keratoconjunctivitis of the moraxellosis etiology; it was undetected in Kazakhstan previously. Therefore, the disease was not widespread among local cattle, hypothetically due to the isolated rearing practices of imported livestock.

The literature presents eye disease descriptions characterized by the following symptoms: Severe lacrimation, photophobia, conjunctival edema, opacity in the center of the cornea, and ulceration. Authors conditionally distinguished between three stages of conjunctivitis, smoothly turning into each other [10,14-16]. On average, 25-30% of the sick remain blind or lose 50% or more of their sight. In 1979, in Australia, losses due to reduced production profitability reached $22 million, including treatment of affected eyes, which cost $1.5 million [17].

Until recently, there was a contradictory view on the pathogenesis and etiology of massive eye diseases. Some researchers have isolated *M. bovis* and *Mycoplasma bovoculi* during outbreaks. Others detected infectious rhinotracheitis (IRT), rickettsia, and nematode viruses of the *Thelazia* genus. However, there is no clear correlation between IBK symptoms and mycoplasmas. In some cases, the IRT virus can cause acute conjunctivitis, but the inflammatory process does not progress to ulceration and severe opacity, as is often the case with IBK. The literature includes reports of simultaneous isolation of *Chlamydia psittaci* and *M. bovis* in IBK [18]. A current general idea has formed about the leading role of the occurrence of this disease belonging to the *M. bovis* bacterial species. According to a group of researchers at the US National Animal Disease Center (Ames), a virulent strain of *M. bovis* can invade the epithelial cells of the cornea of the eye in cattle and causes keratitis in the absence of predisposing factors [19].

Therefore, the need to conduct scientific research on moraxellosis is associated with the great damage caused to livestock, as well as the loss of live weight in animals, the receipt of non-viable young animals, a decrease in the rate of reproduction, and high costs of conducting health-improving and preventive measures. Similarly, moraxellosis infection tends to spread. Therefore, the total damage to business entities increases. For a successful fight, one should monitor studies on the extent of the spread of infectious keratoconjunctivitis and the incidence dynamics in different sexes and age groups, including various breeds of farm animals. Monitoring the prevalence and incidence of outbreaks is important for implementing therapeutic effects to determine the staging and seasonality of manifestation of the clinical signs of this disease. Furthermore, with a preventive purpose and the implementation of effective health-improving measures, it is necessary to disclose the transmission mechanisms of the disease pathogen. It is vital to establish its biological properties, pathological processes, and immunological tests to determine the immunological status of animals concerning moraxellosis infections.

This study aimed to clarify the epidemiological (epizootic) situation of moraxellosis in cattle from Kazakhstan. This investigation includes studies on the incidence in various age and sex groups of the animals, the incidence in individual herds, and the number of unfavorable epidemiological (epizootic) groups. We also visualize individual quantitative and qualitative indicators of the epidemiological (epizootic) process (the staging of the disease’s development, its influence on the contamination of transmission factors of the pathogen, and the infection of possible carriers of the infectious origin). We visualize the mechanism of pathogen transmission, the pathway of *Moraxella*’s penetration into the bodies of healthy animals, and its immunological reactivity manifestation. From the obtained results and implementation of veterinary and sanitary measures, allowance for developing more effective antiepizootic measures will be made possible.

## Materials and Methods

### Ethical approval

The experiments and the methods used for conducting research on laboratory animals comply with the biological safety requirements and ethical principles of experimentation on animals set forth in the European Convention for the Protection of Vertebrate Animals used for Experimental and Other Scientific Purposes. The research protocol was discussed and approved at a meeting of the local ethical committee of the Kazakh Scientific Research Veterinary Institute of the Science Committee of the Ministry of Education and Science of the RK dated August 29, 2017.

###  Study period and location

The study was carried out from January 2018 to December 2020 in Almaty region of the Republic of Kazakhstan.

### Animals

This study investigated the epidemiological (epizootic) prevalence of moraxellosis in different breeds, ages, and sex groups of cattle in epidemiological (epizootic) units of some of the RK’s economic entities (EEs). This study was conducted based on the data of veterinary reports and personal studies (2016-2020). Simultaneously, we clinically examined both imported and local cattle of various age groups and breeds (Aberdeen Angus, Hereford, Holstein Friesian, Kazakh white-headed, Auliekol, and local outbred animals) on farms. We also conducted bacteriological studies of pathological biomaterial taken from the animals’ affected eyes.

In a clinical study of cattle for infectious keratoconjunctivitis, we noted the animal’s general condition, the presence (absence) of lacrimation, photophobia, hyperemia of the conjunctival vessels, blepharospasm, iridospasm, seromucous or seropurulent discharge from the eyes, opacity, and (or) ulceration of the cornea.

### Laboratory research methods

The study of the biological properties of the isolated cultures of *Moraxella* and the selection of the most promising of them for the manufacture of immunobiological preparations were carried out in strict accordance with the sanitary rules “Safety of work with microorganisms of I — II pathogenicity groups,” Code of Practice No. 13 dated January 10, 2012.

To study the biological properties of isolated cultures, we collected samples of pathological material from animals with further clinical signs of keratoconjunctivitis from the surface of the third eyelid of the affected eyes. These samples were taken with sterile swabs placed in a plastic handle with a 12 mm×150 mm transport tube and transport medium (Amies medium) in individual packages (sterile) (China). The whole setup was then placed into a Thermos flask filled with ice for transport to the laboratory. In total, 1425 samples of biomaterial from 475 animals were subjected to bacteriological research.

Our laboratory investigations complied with the “Methodological recommendations for laboratory diagnosis of infectious keratoconjunctivitis of cattle caused by *Moraxella bicelli*,” approved by the Bureau of the Veterinary Medicine Department, Russian Academy of Agricultural Studies [20]. The studied cultures were identified by morphological, tinctorial (tinctorial properties are the ability to react with dyes and color in a certain way), cultural, and enzymatic (biochemical) properties, as well as by immunological reactions with positive serum.

For this purpose, we prepared smears from each sample of pathological material. Then, we stained according to Gram’s method and examined the samples under a microscope with an immersion system, noting the presence or absence of morphology of microflora in them. Then, we inoculated the material on Hottinger’s agar, consisting of enzymatic peptone (15 g/L), sodium chloride (6.5 g/L), yeast extract (0.5 g/L), and tryptophan (0.0015 g/L), adding 5-6% defibrinated blood of small ruminants. The results were then taken into account after 12-24 h of incubation at 37°C and inoculating colonies typical for *Moraxella* and other types of colonies on fresh nutrient media to isolate pure cultures and later to identify them. To study the morphological properties, we stained smears from cultures grown on a nutrient medium according to Gram’s method and examined them under a microscope.

### Enzymatic properties

Gelatin liquefaction was used to determine the proteolytic activity. For this purpose, we prepared a 0.5% solution of gelatin, sterilized the medium, and added 5% of the blood turnover from the cattle before inoculation. The deep injection was used to perform the inoculation addition into a gelatin column. We incubated the inoculations at room temperature (20-22°C) and registered the result daily for 7 days. If the microbe did not liquefy in gelatin, the medium retained a dense consistency, as in control, without growing. In the presence of gelatinase, the medium liquefies.

We conducted the litmus milk test with low-fat milk and a litmus tincture. To prepare the litmus infusion, we took 5 g of dry litmus, rubbed it into a powder in a mortar, transferred it to a bottle with a capacity of 100.0 cm^3^, and poured 50.0 cm^3^ of ethyl alcohol into the bottle, which was changed daily. On the 4^th^ day, we drained the alcohol, dried the litmus in a thermostat, and placed it in a flask. The alcohol was then dissolved in 50.0 cm^3^ of distilled water when heated and filtered. Then, we prepared a litmus medium with milk by adding 5-10% of the litmus infusion to skim milk and the same amount of 10% solution of sodium bicarbonate. The prepared medium was then poured into tubes and autoclaved at 0.5 atm for 30 min. The litmus milk was also inoculated with a loop of the test bacteria.

We studied carbohydrate fermentation by seeding 2-3 drops of the *M. bovis* broth culture into test tubes containing 5% Hottinger whey broth and 0.5% carbohydrates (arabinose, glucose, maltose, rhamnose, mannitol, sucrose, raffinose, galactose, sorbitol, and dulcite). The inoculations were incubated at 37°C for 24 h. The test for catalase activity was conducted by applying 0.1-0.3 cm^3^ of freshly prepared 3% hydrogen peroxide with a pipette to the colony grown on blood Hottinger’s agar during the day. The foam formation was then evaluated as a positive test for catalase activity. To determine the ability to form indole, we placed filter papers soaked in a saturated aqueous oxalic acid solution under the lid of an inoculated test tube with hot Hottinger’s broth. The filter paper was then placed at 1.0-1.5 cm above the surface of the medium. If indole was present after 24 h of cultivation at a temperature of 37°C, the lower end of the paper had a blue or lilac coloration 0.3-0.5 cm downward. The color did not change if indole was absent.

We used the complement fixation test (CFT) for serological diagnostics. This diagnostic test is two-staged. The first stage aimed to bind the known antigen and the target antibody with help from the guinea-pig complement. The second was an indicator system to detect the remaining (unbound) complement in the first phase in the absence of the desired antibodies. This indicator phase of the reaction was conducted by introducing it into the first phase of the hemolytic mixture. This mixture consists of the corresponding volumes of a 3% suspension of erythrocytes and hemolytic serum in a triple working titer. The hemolysis of erythrocytes in this mixture can only be achieved with a complement. If the complement was unbound in the first phase, erythrocyte hemolysis occurred in the second phase; that is, the antibody detection reaction was negative [21]. For its implementation, we used the following components: antigen, antibodies, and complement (the first system), then sheep erythrocytes, and hemolytic serum (the second system).

We also lysed cultures of killed *Moraxella* microorganisms using a low-frequency ultrasonic disperser, which served as an antigen for CFT. Table-1 describes the identification of the reference and isolated strains.

**Table-1:** Comparison of the main biological properties of the isolated cultures of *Mycobacterium bovis* with a differentiated culture in the Federal State Budgetary Institution, Kazan.

Properties	Studied cultures											Reference culture

	1	2	3	4	5	6	7	8	9	10	11	
Hemolysis on blood agar	±	−	+	+	+	+	+	+	+	±	+	+
Acetate recovery	±	−	−	−	−	±	−	−	−	−	−	−
Nitrate reduction	−	−	−	−	−	−	−	−	−	−	−	−
Gelatine liquation	+	−	+	−	+	+	±	+	+	+	+	+
Litmus milk coagulation	+	+	+	+	+	+	+	+	+	+	+	+
Carbohydrate fermentation	−	−	−	−	−	−	−	−	−	−	−	−
Catalase activity	+	+	+	+	+	+	+	+	+	+	+	+
Indole formation	−	−	±	−	−	−	−	−	−	−	−	−

“+” is a positive result; “−“ is a negative result; “±” means that the result varies

## Results

Monitoring of the IBK disease in cattle from the territory of the RK shows that one of the main reasons for the appearance of the disease was the import of breeding stocks, among which there were sick animals. Therefore, the movement of infected animals across RK regions without appropriate antiepizootic measures led to a widespread disease outbreak and more disadvantaged farms.

We previously registered the first cases of the disease in the livestock of cattle in July during an epizootological survey of the livestock for meat production. This investigation was conducted at Bayserke-Agro LLP on the Zhamantal pasture land in 2017. The disease first manifested as conjunctival edema and lacrimation, which covered 5.3% of the livestock. At the initial stage, there was a serous outflow from the medial corner of the eye, and a little later, an accumulation of mucous and purulent exudate occurred. Palpation revealed soreness of the eyelids and an increase in local temperature. Subsequently, a pronounced corneal opacity appeared; on the 6^th^ to 10^th^ days, erosion with a diameter of about 1 mm developed in its center, which soon turned into an ulcer.

The ulceration stage was accompanied by strong anxiety of the animals, including a relatively high body temperature reaching up to 41°C, and a refusal to feed. The corneal opacification then spread rapidly in all directions from the ulcer. Over the next 10-15 days along the edge of the lesion, the vasculature development appeared. In some, especially severe cases, it surrounded the entire cornea along the periphery, forming a red rim. These changes led to the thickening of the cornea and loss of its transparency. Cases when the vessels grew to the center of the cornea and formed a nipple-shaped eminence also occurred. Subsequently, the blood supply was cut off, and the bright color of the plexus of the vessels took on a pale shade. Within 25-50 days, vascular consolidation decreased in size and completely disappeared. Eyeball deformation occurred among animals, especially young animals at 6-10 months of age. In 2% of calves at 6-8 months of age, all corneal layers were perforated because of its ulceration, and the vitreous was leaking out, leading to unilateral or bilateral blindness. The lesion occurred in one eye, and if in both, then at different stages of the course. We noted these animal disease features when examining cattle (mainly the Aberdeen Angus breed) in the southern and in the northern and eastern zones of the RK. Confirmation of observed symptoms was by clinical manifestations of the disease.

In trying to discover the alleged source of the pathogen and possible ways of tackling the disease, we found that the appearance of the disease in animals was associated with importing Aberdeen Angus cattle from abroad (Canada, Australia). This breed contained some sick animals with clinical signs of eye damage. During the research, we isolated the *M. bovis* pathogen. The epizootic process intensified in summer, which we associated with pathogen vectors, such as stinging insects. Increased pathogenicity resulted in the pathogen’s passage when transmitted from an infected animal to a healthy one.

Observations established that the disease pathogen could use transmission factors (non-living objects), while insects were probable carriers. Additional factors influencing the course and clinical manifestation of the disease were eye injuries, hot weather, wind, and dust. The movement of infected cattle that carried the pathogen of the disease from one farm to another was another proposed direct path to a significant spread of the disease. Consequently, the continuity of the epizootic process was ensured, which led to the appearance of new outbreaks of the disease.

**Figure-1:**
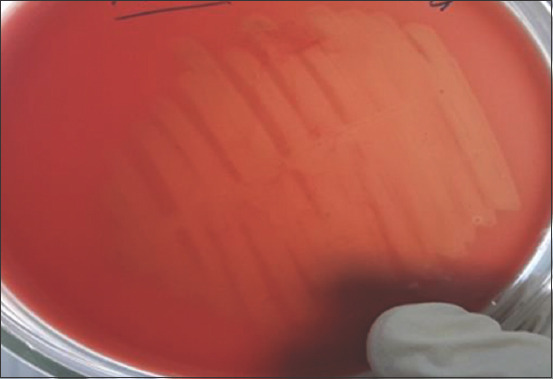
Growth on a nutrient medium with the addition of up to 10% of defibrinated blood of the studied culture (hemolysis zone).

Thus, to maintain the welfare of animals in the RK from moraxellosis, comprehensive monitoring of the epizootic situation was used to identify the main risks of the probable appearance and spread of the disease among cattle. Due to studying the morphological properties of the culture, Moraxella colonies were characterized as flat, round, gray and white, and indented formations with a 1-3 mm diameter. Those with loose consistency and those surrounded in most cases by a narrow (0.5-1.0 mm) hemolysis zone were characterized as well (Figure-1).

**Figure-2:**
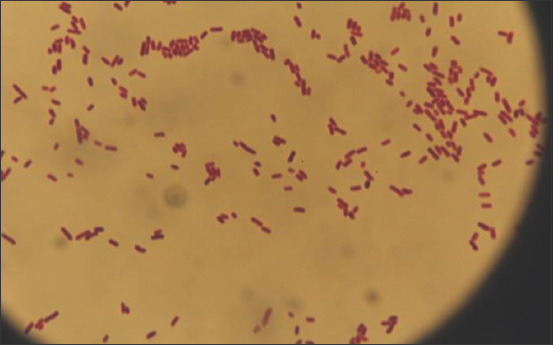
Culture of isolated bacteria from affected cow eyes (staining according to Gram’s method).

After staining according to Gram’s method, identical Gram-negative microorganisms were found, represented by bacteria with a diameter of 0.6-1.0 μm with characteristic paired forms and more articulations. Such colonies were characteristic of *M. bovis*. Therefore, we made smears from 2 to 3 of such colonies and then stained them according to Gram’s method. Then, we studied them under a microscope. Figure-2 shows Gram-negative, short, thick, rounded-edged bacteria.

In the presence of Gram-negative, polymorphic, short up to cocci, thick with rounded edges of bacteria located singly, in pairs, or the form of short chains, we made inoculations from these colonies on a Hottinger’s agar. They caused changes in the litmus milk characteristic of the pathogen. In the conditions of erobiosis, they caused the milk to alkalize. The upper layer of the litmus milk with a height of up to 0.5-1 cm turned dark blue, the medium layer turned light blue, and the lower layer turned white with grains of peptonized milk. Gram-negative short thick rods with rounded ends arranged in pairs or short chains represented the reference strains of the bacteria *M. bovis*. Table-1 shows the biochemical analyses of the culture under study and their reference strains (Federal State Budgetary Institution [FSBI], Kazan).

For identification, pure cultures were separately inoculated into test tubes with appropriate media for determining saccharolytic properties (Hiss’s media), as well as catalase activity. We used Giss’s medium to differentiate microorganisms by their ability to ferment sugars. The indicator of the medium is bromocresol purple. During carbohydrate fermentation and the release of acid, it changes the color of the medium. The data in Table-1 testifies that the main biological properties of the isolated cultures corresponded to the one identified in the Kazan FSBI as *M. bovis*.

In Hiss’s media, the A20 culture being studied had a fermenting effect on mannitol and glucose, with a color change to yellowish and gas formation. There was no fermenting effect on sucrose and sorbitol (the color did not change, and there was no gas) observed. The mouse testicular fragment (MTF) culture also had a fermenting effect on glucose and sucrose, with a color change from blue-green to yellowish without gas formation, and no fermenting effect on mannitol and sorbitol. Alternatively, the K-136 culture had a fermenting effect on mannitol and glucose, with a color change to yellowish with gas formation and no fermenting effect on sucrose and sorbitol.

In Hiss’s media, the *Moraxella* bacteria cultures had no fermenting effect on sugars. The control tubes, therefore, remained unchanged. Thus, to determine the fermentation of catalase, 1-2 mL of 1% hydrogen peroxide solution was poured onto the surface of a 24-h culture of the referenced bacterial cultures under study on a slant meat infusion agar. All studied bacterial cultures were catalase-negative. Furthermore, to study the antigenic properties and determine the immunological relationship of the isolated cultures of microorganisms named A-20, MTF, and K-136 with respect to the reference strains, we performed serological tests.

The agglutination reaction on the glass showed that all and control cultures studied gave weak agglutination with specific moraxellosis hyperimmune rabbit blood-sera. The *M. bovis* species were thus classified as bacteria with the morphological and tinctorial properties described above. They were also characterized under the conditions that they were inactive, did not grow on simple agar or broth, did not recover sodium acetate, did not reduce nitrates to nitrites, did not ferment carbohydrates, did not produce indole and liquefied gelatin, and possessed catalase activity.

We also conducted epizootological monitoring of cattle moraxellosis, in EE of the RK. Similarly, we identified the features of the occurrence, development, and manifestation of the disease and established the degree of its spread. It is from Table-2, cattle moraxellosis was recorded in many epizootological units of the RK. Of the 475 animals studied, 72 responded positively by CFT, which was 15.1%, while the pathogen was isolated from 128 animals, or 26.94%. The moraxellosis pathogen was isolated from all animals positively reacting to CFT, indicating its specificity. Comparative analysis of the data of the Meat Union of Kazakhstan for the period of 2012-2018 (39.98%) and the results of our studies (26.94%) also indicate a decrease in the incidence of moraxellosis in animals by 32.6%, which is associated with the effectiveness of special veterinary measures to combat this disease [22].

**Table-2:** Information on the distribution of moraxellosis keratoconjunctivitis among cattle in the Republic of Kazakhstan based on the results of our research.

Name of the region	Number	Number of animals studied	Reacted positively to the complement fixation test		The pathogen to the number of sick animals among the studied animals was determined	
	
			Absolute quantity	%	Absolute quantity	%
Almaty	2	26	2	7.69	10	38.46
Karaganda	4	74	16	21.6	39	52.7
Zhambyl	3	39	1	2.56	2	5.12
Akmolinsk	3	39	3	0.76	8	20.51
Turkistan	3	39	6	15.4	10	25.64
Aktobe	2	25	0	0	0	0
West Kazakhstan	3	39	0	0	0	0
Kostanay	4	52	31	59.6	41	78.84
Pavlodar	3	39	3	7.69	3	7.69
North Kazakhstan	3	39	6	15.4	10	25.64
Kyzylorda	2	26	0	0	0	0
East Kazakhstan	3	38	4	10.5	5	13.15
Total:	35	475	72	15.1	128	26.94

**Figure-3:**
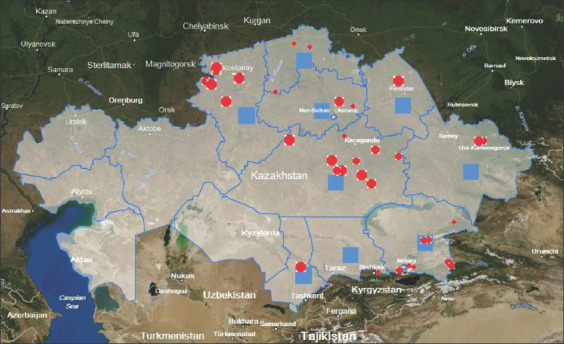
Epizootic visualization map with quantitative and qualitative indicators of the epizootic process of moraxellosis in the context of regions of the Republic of Kazakhstan in 2019 [Source: www.maps.google.com].

**Figure-4:**
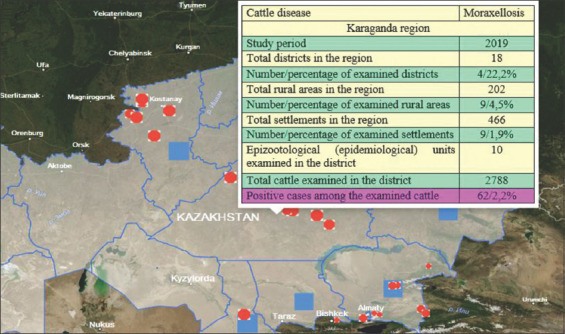
Details of the epizootic visualization map data with quantitative and qualitative indicators of the epizootic process of moraxellosis using the example of the Karaganda region in 2019 [Source: www.maps.google.com].

**Figure-5:**
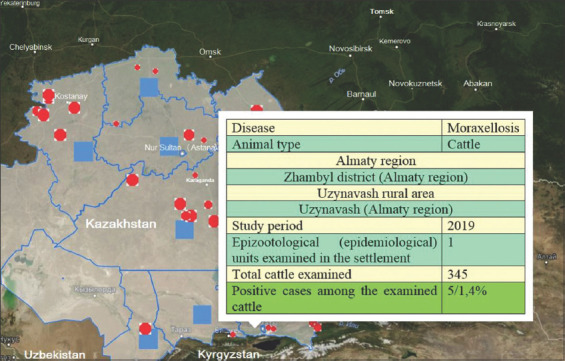
Detailing the data of the epizootic visualization map with quantitative and qualitative indicators of the epizootic process of moraxellosis on the example of the Uzynagash rural district of the Almaty region in 2019 [Source: www.maps.google.com].

Based on the results of the studies, epizootic maps were developed (Figure-3) for visualization with qualitative and quantitative indicators of the epizootic process in various regions of the RK for cattle moraxellosis in 2019. Figures-4 and 5, which show detailed epizootic data characterizing the degree of infection spread in the context of regions, districts, rural districts, and EEs, show the level of moraxellosis in the cattle population, as well as the relative and absolute indicators of animal morbidity.

Eye diseases in cattle with moraxellosis etiology were registered in 2019 in many epizootological units of EEs in the RK. Thus, out of the total presence of 32,710 animals, the disease was detected in 832 cases (2.54%). The given data are quantitative indicators of the epizootic process. In the EEs of the RK, where cattle were imported from abroad, disease presence was revealed in all cases. There were also links to the epizootic process, that is, the source of the disease pathogen, the infectious principle transmission mechanism (transmission factors such as inanimate objects and carriers such as insects), and susceptible animals. Registration of animal diseases regardless of quantitative characteristics (morbidity, prevalence, incidence, proportion in the presence of other diseases, etc.) is a qualitative indicator of the epizootic process.

Thus, the eye disease in cattle of moraxellosis etiology is observed in all regions of the RK where breeding animals were imported, mainly belonging to the Aberdeen Angus breed. Simultaneously, the incidence reaches 27%. Clinical examination of animals was used to diagnose moraxellosis in cattle and by CFT in the study of blood serum. Simultaneously, we found that the CFT’s sensitivity was inferior to the bacteriological method due to the absence of specific antibodies in the blood serum where the pathogen of the disease is not detected. Clinical signs of the disease began to appear more in the spring, reaching their peak in August and September. The isolated cultures of the pathogen in their biological, biochemical, and immunological properties are also identical to the reference strains differentiated in FSBI, Kazan.

## Discussion

We first identified the lesions in the eyes of cattle caused by moraxellosis in the southeast of Kazakhstan among meat production animals of the Aberdeen Angus breed. The animals were among the ones imported from foreign countries (such as Canada and Australia). Earlier, according to the veterinary reporting data, this disease among the specified animal species in Kazakhstan had not been registered. Observations showed that eye damage was noted only among animals up to 2 years of age of this breed.

In a herd of cattle, meat production animals of the Aberdeen Angus and Hereford breeds were kept together. Simultaneously, eye damage was noted only in animals of the Aberdeen Angus breed. There are reports of a predominant eye lesion of moraxellosis etiology in Hereford cattle. The data we obtained on the greater damage to the eyes of the Aberdeen Angus cattle caused by moraxellosis do not agree with other researchers’ data. Our result is due to the peculiarities of the pathogen clone circulating in this region or the reduced resistance of the organism of different age groups of the Aberdeen Angus animals.

There were also data on susceptibility to the disease and cattle of other animal species in the special literature, particularly sheep, goats, camels, pigs, and poultry [23]. However, our epizootological observations indicate the absence of moraxellosis lesions among other animal species kept together with the affected group of livestock. The clinical signs of the disease we noted and the stages of development of the pathological process coincide with the available data in the literature [17,20,24-26]. This result does not raise doubts about the reliability of the data obtained.

We noted that the disease manifested during the spring-summer period, but the most clinically sick animals and disease severity occurred during the summer-autumn period. This observation was associated with the onset of flight and its subsequent suspension of stinging insects as possible carriers of the infection. Bacteriological studies of insects, we caught from the affected eyes of cattle showed their participation in transferring the pathogen of the disease. These data indicate the need for appropriate insecticidal measures.

The development of antiepizootic measures requires monitoring studies for diseases among cattle and other animal species in separate epizootic units. This study was the first to map the epizootic situation with quantitative and qualitative indicators of infection for individual epizootic units. Such data facilitate the development of antiepizootic measures and procedures to implement preventive and recreational measures. The isolated cultures of the pathogen in their biological, biochemical, and immunological properties, in particular, the characteristic growth on Hottinger’s blood agar, recovery (or reduction) of sugars, tinctorial, and morphological properties, were also identical to the reference strains.

We were also the first to reveal the induction of complement-binding substances in parasitic *Moraxella* cattle eye infection. However, we found that the bacteriological method for identifying the pathogen was superior to the named immunological test. Simultaneously, in all cases, *Moraxella* appeared in animals with a positive result for the CFT. This result indicates the specificity of this diagnostic method’s indications that require studying the sensitivity and specificity of other immunological methods.

Thus, our study, to a certain extent, reveal the epizootic chain in moraxellosis in cattle, in particular, the source of the pathogen of the infection (sick animals), the mechanism of transmission of the pathogen of the disease (stinging insects and contact route), and the presence of the most sensitive and susceptible animals (cattle of a certain breed and age group).

## Conclusion

During the clinical examination of cattle, we found animals with eye lesions and clinical manifestations such as the presence (absence) of tearing, luminosity, hyperemia of the conjunctival vessels, blepharospasm, iridospasm, seromucous or seropurulent discharge from the eyes, and corneal opacity and (or) ulceration. Biomaterials were selected from animals with clinical manifestations of the disease for research on moraxellosis. The study established the epizootic situation in the EE of the RK on moraxellosis among cattle in the context of epizootic units.

According to the data of the conducted studies, we clarified the epizootic situation of cattle moraxellosis in the RK. We developed epizootic visualization maps with quantitative indicators of the epizootic process in various regions of the RK for cattle moraxellosis in 2019. The data obtained from studies of the properties of the isolated cultures compared with the characteristics of the reference strains, therefore, gave grounds to classify them as belonging to the *Moraxella* genus.

## Authors’ Contributions

NPI and FAB: Conception and design, drafting the article, and revising it critically. AMN: Acquisition of data. RSS: Conception and design, analysis and interpretation of data, and drafting the article. BZI: Acquisition of data, analysis and interpretation of data, and drafting the article. NZA: Acquisition of data and analysis and interpretation of data. All authors read and approved the final manuscript.
